# Computational evidence that fast translation speed can increase the probability of cotranslational protein folding

**DOI:** 10.1038/srep15316

**Published:** 2015-10-21

**Authors:** Ercheng Wang, Jun Wang, Changjun Chen, Yi Xiao

**Affiliations:** 1 Biomolecular Physics and Modeling Group, Department of Physics, Huazhong University of Science and Technology, Wuhan 430074, China

## Abstract

Translation speed can affect the cotranslational folding of nascent peptide. Experimental observations have indicated that slowing down translation rates of codons can increase the probability of protein cotranslational folding. Recently, a kinetic modeling indicates that fast translation can also increase the probability of cotranslational protein folding by avoiding misfolded intermediates. We show that the villin headpiece subdomain HP35 is an ideal model to demonstrate this phenomenon. We studied cotranslational folding of HP35 with different fast translation speeds by all-atom molecular dynamics simulations and found that HP35 can fold along a well-defined pathway that passes the on-pathway intermediate but avoids the misfolded off-pathway intermediate in certain case. This greatly increases the probability of HP35 cotranslational folding and the approximate mean first passage time of folding into native state is about 1.67μs. Since we also considered the space-confined effect of the ribosomal exit tunnel on the cotranslational folding, our simulation results suggested alternative mechanism for the increasing of cotranslational folding probability by fast translation speed.

Nascent peptides start folding within the ribosomal exit tunnel during their syntheses^1^,^2^ and this is called as cotranslational folding^3^,^4^,^5^,^6^,^7^,^8^,^9^,^10^,^11^,^12^. The cotranslational folding process is controlled by translation speed and special ribosomal structure^2^,^13^,^14^,^15^,^16^. Experimental and theoretical studies on the cotranslational folding were highlighted and novel phenomena were observed^7^,^16^,^17^,^18^,^19^,^20^,^21^,^22^,^23^,^24^,^25^,^26^,^27^,^28^,^29^,^30^. For examples, it was found that the synthesized peptides take on definite secondary structures while they are growing on the ribosome^2^,^16^. There are experimental evidences showing that decreasing the codon translation rate can increase the probability of cotranslational folding of some proteins^14^,^24^,^31^. Recently, a kinetic modeling indicates that fast-translating codons can also increase the probability of cotranslational protein domain folding by avoiding misfolded intermediates^26^. In this paper we provide computational evidence for this phenomenon.

The villin headpiece subdomain HP35 (PDB id: 1yrf)^32^ is an ideal model to elucidate the phenomenon studied here. Firstly, HP35 is a small and fast folding protein of 35 residue and with *in-vitro* folding rate of about 4.3μs (experiment)^33^ and 5.6 ~ 8.2μs (simulations^34^) and so we can simulate the entire folding process by using all-atom molecular dynamics. Secondly or most importantly, HP35 has both off-pathway and on-pathway intermediates^32^. The off-pathway intermediate is characterized by the formation of N-segment (residues 3 to 21, helix I and helix II) and the on-pathway intermediate is characterized by the formation of C-segment (residues 15 to 33, helix II and helix III). The off-pathway intermediate can never directly fold into the native sate and is a misfolded state. Furthermore, it is unstable without the stacking interactions with the C-segment through the hydrophobic core residues^35^,^36^ and so its stability non-monotonically changes with nascent chain length. Therefore, HP35 is an ideal model to see if fast translation can avoid the misfolded off-pathway intermediate during cotranslational folding.

The cotranslational folding of HP35 is simulated in three steps: (1) folding in the ribosomal exit tunnel, (2) co-releasing folding from the tunnel and (3) free folding in bulk. This not only considers the effect of co-translation on the folding but also that of the ribosomal exit tunnel that occurs during *in-vivo* folding of nascent peptides^2^,^16^. In the first step of the simulations, HP35 folds starting from an extended conformation in a model of the ribosomal exit tunnel. For this step the results of our previous work^16^ will be used and it was found that HP35 forms native α-helices quickly in the tunnel (Supplementary Figure S1). After folding in the tunnel, in the second step, HP35 is released into bulk from the tunnel consecutively one by one residue at a given time interval. Here, we select the last conformation of HP35 in the first step as the starting structure for the second-step simulations. In this conformation almost all the native α-helices have formed but the chain is still extended (Supplementary Figure S1)^16^. In the third step, the structure just when all the residues are released from the tunnel HP35 is simulated in bulk.

To see if fast translation speeds can increase the probability of cotranslational folding by avoiding misfolded intermediates^26^, in the second step of the simulations, we use translation speeds of one residue per 2ns and 6ns, respectively, which are much faster than the synthesizing rate (about 4 and 20 residues per second in eukaryotes and prokaryotes, respectively^13^,^37^). Using such fast translation speeds is due to the limitation of current computing capability although they are difficult to realize in practice. However, the effective translation speeds may be slower since in the simulations we do not consider the interactions between the nascent peptide and the ribosome that can greatly slow down the folding process of the nascent peptides^1^. Therefore, our simulations can give some insights into the behaviors of cotranslational folding. The simulated folding rate of HP35 comparable to the measured *in-vitro* folding rate also indicates this (see the following). Furthermore, we have also used an approximation that all the residues (codons) are translated in the same speed.

For the translation speed of one residue per 2ns, it is found that, just when all the residues are released from the tunnel, HP35 is already loosely packed and has conformations with similar RMSD (Root Mean Square Deviation) values (about 6Å) relative to the experimental structure in all of the five simulations (Supplementary Figure S2). Then, during the folding in bulk, four of the five simulated trajectories reach native conformations (when the RMSD is less than 2Å) within 3μs and two with sub-angstrom RMSD (about 0.30Å). The trajectory not leading to the folded state within 3 μs (T2-5) has already reached the on-pathway intermediate with a stable conformation (RMSD = 3.45 Å) that the position of helix I is at the opposite side of the plane formed by helix II and helix III in relative to that in the folded state^32^(see Supplementary Figure S3). The mean first passage time into native state is about 1.67μs approximately calculated based on the those of the four folded trajectories and by setting the first passage time of the unfolded trajectory as 3μs and it is much faster than the *in-vitro* folding rate (about 4.3 μs (experiment)^33^ and 5.6 ~ 8.2 μs (simulation)^34^). The first passage times of the four folded trajectories are 0.19 μs, 1.41 μs, 1.55μs and 2.21 μs, respectively.

For the translation speed of one residue per 6ns, the conformations just when all the residues are released from the tunnel are more extended and have RMSD values of about 9Å in all of the five simulations (Supplementary Figure S2). During the folding in bulk, only three of the five simulated trajectories reach native conformations within 3μs. The mean first passage time is about 2.15μs based on the three folded trajectories and by setting the first passage time of the two unfolded trajectory as 3μs (Supplementary Figure S2). In this case the mean first passage time is longer than that for the translation speed of one residue per 2ns.

To understand the results above, we built the folding free energy landscapes from the five trajectories for each translation speeds (Fig. 1). Figure 1a shows that, for translation speed of one residue per 2ns, the folding is almost along a well-defined pathway with the C-segment folding into native state first and then the N-segment, i.e., HP35 has passed the on-pathway intermediate but avoided the off-pathway intermediate^32^, as expected by the kinetic modeling^26^. However, for the case with a translational speed of one residue/6ns, the cotranslational folding has sampled the region of the off-pathway intermediate (Fig. 1b). The reason for this is that in one of the simulated cotranslational folding trajectories (T6-1 in Supplementary Figure S2) the formed helix II have unfolded when the entire chain was released from the tunnel. Especially, in this trajectory, the native contacts of the linker region around Pro21 between helices II and III only form partially (Supplementary Figures S4) and this may decrease the stability of helix III because this linker region plays a crucial role by restricting the movement of the two helices and in stabilizing the native structures and is the initiation site of the folding of C-segment^32^,^38^. In fact helix III indeed has partially unfolded during folding in bulk (Supplementary Figure S3) and this makes HP35 fold into the misfolded intermediate. In other simulated trajectories that helix II unfolded, Pro21 forms the native contacts completely and so HP35 folds into the on-pathway intermediate, although it may not fold into the native state within 3 μs (Supplementary Figures S4 and S5).

The folding efficiency can also be shown by using two parameters that describe the tertiary interactions. One is the ratio Q_nat_ of long-range native contacts in the snapshot structure to all native ones. Another parameter is the ratio Q_non_ of long-range non-native contacts to all native contacts. The long-range contacts are defined by the Cα-Cα distances between residues being lower than 7 Å and distances along the sequence larger than three residues. The ratios Q_nat_ and Q_non_ for HP35 are shown in Fig. 2, respectively. Figure 2 indicates that the cotranslational folding with translation speed of one residue per 2ns forms more native contacts and less non-native ones than that with translation speed of one residue per 6ns. Therefore, the former can make the protein fold more efficiently than the latter. But the both cases form less non-native contacts than *in-vitro* folding^16^.

Our results show that the fast translation rate can indeed increase the probability of HP35 folding by passing the on-pathway intermediate but avoiding the misfolded off-pathway intermediate in some cases, as predicted by the kinetic modeling^26^. Since the *in-vivo* folding rate for HP35 is unavailable we cannot compare our results with it but the simulated folding for the translation speed of one residue/2ns are indeed along the most efficient pathway. Here we have also considered the effect of the space confinement of the ribosomal exit tunnel that occurs in practice. Both factors may play roles in making the proteins fold into native states efficiently along a well-defined pathway, as proposed by Levinthal^39^. Furthermore, this leads to an alterative mechanism of increasing the probability of the protein domain folding. The kinetic modeling suggests that the slow translation makes the nascent peptide have enough time to form the off-pathway intermediate if the misfolded core is located in a position close to the N-terminus of the nascent peptide. When the entire chain is synthesized it cannot fold into the native state until the misfolded core first unfolds. On the other hand, the fast translation speed can avoid the formation of the misfolded core close to the N-terminus of the nascent peptide and thus increase the probability of the nascent peptide folding. When considering the effect of the space confinement of the ribosomal exit tunnel, the nascent peptide has already formed the native secondary structure before it is released from the tunnel, at least for the protein domains of mainly α-helical structure, such as HP35 (Supplementary Figure S1). The formed native secondary structures can avoid the formation of the misfolded core in the N-terminus of the nascent peptide if the translation speed is fast. However, if the translation is slow, the formed secondary structures may unfold and in this case the folding is similar to the *in-vitro* folding when the entire chain is synthesized. This increases the probability of the protein folding into the misfolded off-pathway. In practice the translation speeds are much lower than 2ns or 6 ns and the formed secondary structures in the N-terminus of the nascent peptide may be stabilized by the chaperons, such as Trigger factor^1^. Therefore, our results suggest that increasing translation speed may play the role of the chaperons that can increase the probability of protein folding.

## Methods

### Simulation of co-releasing folding and free folding

The cotranslational folding of HP35 is simulated in three steps: (1) folding in the ribosomal exit tunnel, (2) co-releasing folding from the tunnel and (3) free folding in bulk. The method of simulating folding in the ribosomal exit tunnel was described in our previous work^16^. The initial conformation in this work is taken from one of the last structures of the folding trajectories in the ribosomal exit tunnel (Supplementary Figure S1). The simulations of co-releasing folding and free folding in bulk are as follows. In the co-releasing folding process, the initial conformations is aligned along x axis and the residues of HP35 are released from the cylindrical tunnel into bulk with a rate, e.g., one residue/2ns, starting from the N-terminus. In the first 2 ns, residues 2 to 35 are confined in the tunnel while residue 1 can freely fold in bulk, and then in the next 2 ns, residues 3 to 35 are confined while residues 1 and 2 can freely fold in bulk, and so on. In this case it takes 70 ns for HP35 to be released completely from the tunnel into the bulk. To simulate this process, we have implemented a subroutine and incorporated it into AMBER^40^ (“PMEMD” program). After HP35 is released completely from the tunnel, it freely folds in bulk for 3μs.

### Simulation details and parameters

In this work we used two different releasing rates (one residue/2 ns (T2) and one residue/6 ns (T6)) and simulated five trajectories for each case. All simulations had been performed by the Amber 12.0 package with Amber ff12SB force field^40^. The TIP3P cuboid water box model was used and the closest distance between any atom of HP35 and the edge of the box was 12 Å. The system with 3831 water molecules added was equilibrated in the NPT ensemble for 10 ps and all simulations were performed in NPT ensemble with a constant pressure (pres0 = 1.0) at 300K controlled by Langevin dynamics. Two Cl- ions were added in the system in order to ensure the total charge is zero. SHAKE was performed and the bonds involving hydrogen were constrained. The nonbonded cutoffs for Van de Waals and electrostatics interactions was 9.0 Å and electrostatics were calculated using Particle Mesh Ewald (PME) with 84, 48 and 48 grid points along x, y and z axis, respectively. The time step was set to 2 fs and coordinates were saved every 10000 steps.

### Analysis of simulation

The “cpptraj” program in Amber 12.0 package^40^ was used to calculate Cα-RMSDs with the x-ray crystal structure of HP35 as the reference. Cα-RMSDs of the whole protein (residues 2 to 34, excluding terminal residues 1 and 35 owning to the flexibility of terminal residues) were used to evaluate its global folding^32^. Cα-RMSDs of N-segment (residues 3 to 21) and C-segment (residues 15 to 33) are used to generate the free energy landscape. Because of helix II owned by both segments, when N-segment and C-segment are folded, the whole protein folds to native state. The cartoon structures were displayed by VMD^41^. The free energy landscape is calculated using the algorithm proposed by Pande *et al.*^42^, in which the free energy (in *kcal*/*mol*) is defined as -*RT* ln(*N*/*N*_T_), where *N* is the number of conformations of a state of HP35 in the state space defined by two order parameters Cα-RMSDs of the N-segment and C-segment. In practice *N* is calculated as the number of the conformation in each counted region (a small square) with a size of 0.1Å × 0.1 Å in the state space. *N*_T_ is the total number of the conformations in the state space. In this paper the conformations of the state space is from all the five trajectories for each case. Finally, *R* is the gas constant and *T* is temperature.

## Additional Information

**How to cite this article**: Wang, E. *et al.* Computational evidence that fast translation speed can increase the probability of cotranslational protein folding. *Sci. Rep.* **5**, 15316; doi: 10.1038/srep15316 (2015).

## Supplementary Material

Supplementary Information

## Figures and Tables

**Figure 1:**
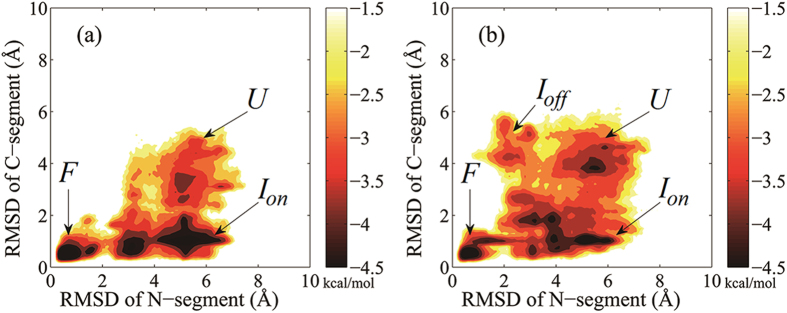
Free energy landscape of HP35 cotranslational folding with translation speeds of one residue per 2ns (**a**) and 6ns (**b**). Each free energy landscape is built from five 3μs simulated trajectories and the order parameters are Cα-RMSDs of the N-segment (residues 3 to 21) and C-segment (residues 15 to 33), respectively. *U* denotes unfolded state, *I*_*on*_ the on-pathway intermediate, *I*_*off*_ the off-pathway intermediate, and *F* the folded (native) state.

**Figure 2:**
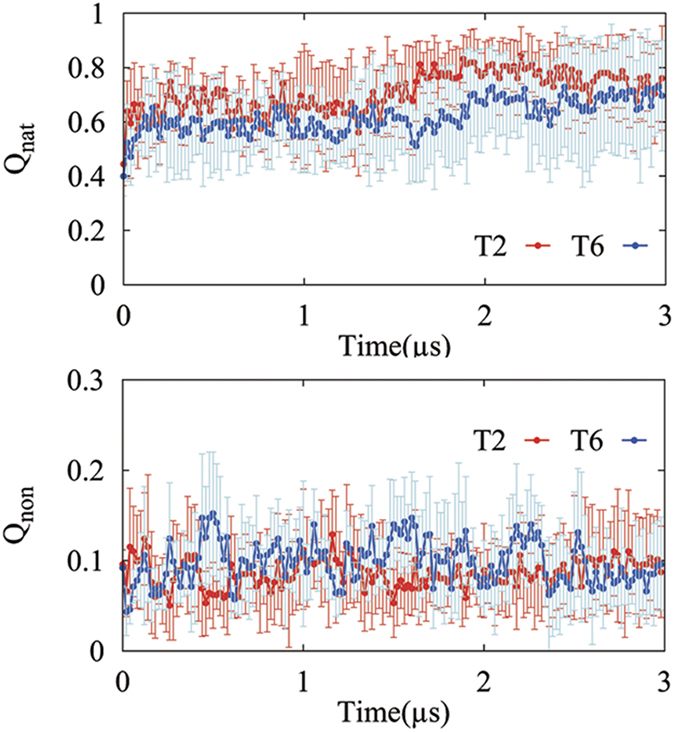
The ratio of long-range native (Q_nat_) and non-native (Q_non_) contacts in the snapshot structures. The error bar is based on the statistical data of five independent simulated trajectories for each case. T2 (red) and T6 (blue) denote the trajectories with the translation speeds of one residue per 2ns and 6ns, respectively.
