# Supplementary material for: Computational evidence that fast translation speed can increase the probability of cotranslational protein folding
Source: Sci Rep. 2015 Oct 21;5:15316. doi: 10.1038/srep15316 (PMC4614103; doi:10.1038/srep15316)
Supplement: Supplementary Information [file srep15316-s1.pdf]

Support Information

Computational evidence that fast translation speed can increase the probability of cotranslational protein folding

*Ercheng Wang, Jun Wang , Changjun Chen and Yi Xiao**

Biomolecular Physics and Modeling, Department of Physics, Huazhong University of Science and Technology, Wuhan 430074, China

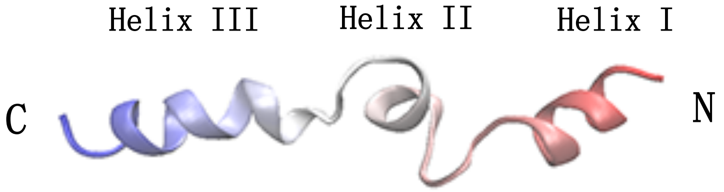

Figure S1. One of the last conformations of HP35 in the simulated trajectories of the folding in the ribosomal exit tunnel. This conformation is selected as the starting structure for the second-step simulations (the co-releasing folding).

* To whom all correspondence should be addressed.

Trajectories	T2-1	T2-2	T2-3	T2-4	T2-5
Just released structure (RMSD)	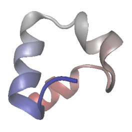 (6.73Å)	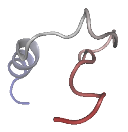 (6.27Å)	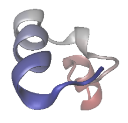 (6.01Å)	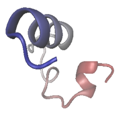 (6.13Å)	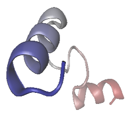 (6.72Å)
First passage time	0.19μs	2.21μs	1.55μs	1.41μs	~

Trajectories	T6-1	T6-2	T6-3	T6-4	T6-5
Just released structure (RMSD)	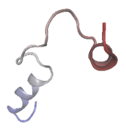 (9.06Å)	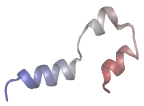 (9.15Å)	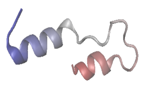 (8.46Å)	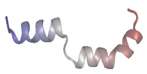 (10.38Å)	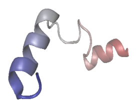 (8.81Å)
First passage time	~	1.05μs	~	2.82μs	0.86μs

Figure S2. Simulation results of cotranslational folding of HP35 starting from the structures just released from the tunnel with a rate of one residue per 2ns (T2) and 6ns (T6), respectively. The RMSD values are relative to the experimental structure of HP35(PDB id: 1yrf). The just released structures are those when all the residues are just released from the tunnel. The first passage time is the time that the protein first folds into the native state, i.e., the RMSD is less than 2Å.

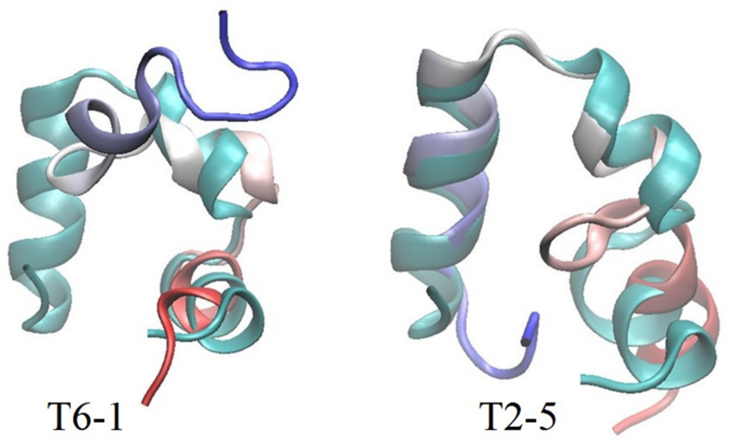

Figure S3. The last structures of HP35 in the two simulated 3μs trajectories (T6-1 and T2-5) superimposed onto the native state (cyan), respectively.

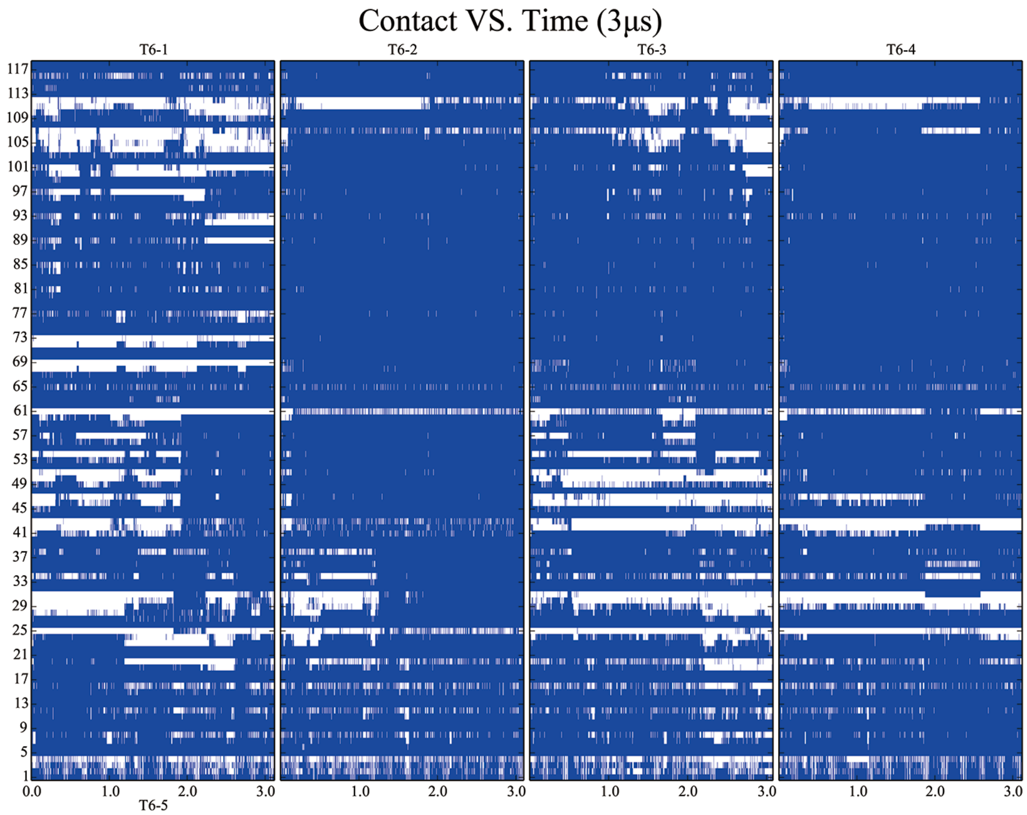

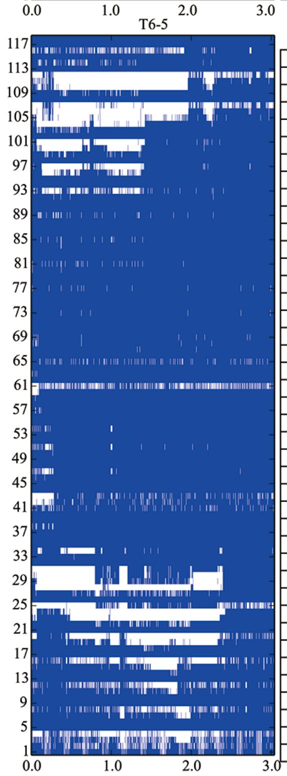

ID	Residue1	Residue2
1	1	2
2	1	3
3	1	5
4	1	6
5	2	3
6	2	4
7	2	5
8	2	6
9	3	4
10	3	5
11	3	6
12	3	7
13	4	5
14	4	6
15	4	7
16	4	8
17	5	6
18	5	7
19	5	8
20	5	9
21	6	7
22	6	8
23	6	9
24	6	10
25	6	13
26	7	8
27	7	9
28	7	10
29	7	11
30	7	12
31	7	13
32	8	9
33	8	10
34	8	11
35	9	10
36	9	11
37	10	11
38	10	12
39	11	12
40	12	13

ID	Residue1	Residue2
41	12	14
42	12	16
43	12	17
44	13	14
45	13	15
46	13	16
47	13	17
48	14	15
49	14	16
50	14	17
51	14	18
52	15	16
53	15	17
54	15	18
55	16	17
56	16	18
57	16	19
58	17	18
59	17	19
60	17	20
61	17	25
62	18	19
63	18	20
64	19	20
65	19	21
66	20	21
67	20	22
68	20	24
69	20	25
70	21	22
71	21	23
72	21	24
73	21	25
74	22	23
75	22	24
76	22	25
77	22	26
78	23	24
79	23	25
80	23	26

ID	Residue1	Residue2
81	23	27
82	24	25
83	24	26
84	24	27
85	24	28
86	25	26
87	25	27
88	25	28
89	25	29
90	26	27
91	26	28
92	26	29
93	26	30
94	27	28
95	27	29
96	27	30
97	27	31
98	28	29
99	28	30
100	28	31
101	28	32
102	29	30
103	29	31
104	29	32
105	29	33
106	29	34
107	29	35
108	30	31
109	30	32
110	30	33
111	30	34
112	30	35
113	31	32
114	31	33
115	32	33
116	32	34
117	33	34
118	34	35

Figure S4. The native contact formation vs. simulation time for HP35 with a translation rate of one residue per 6ns. If a native contact is formed it is marked in blue. The native contacts are numbered from N-terminus sequentially. The residue pair related to each native contact is list in the right bottom.

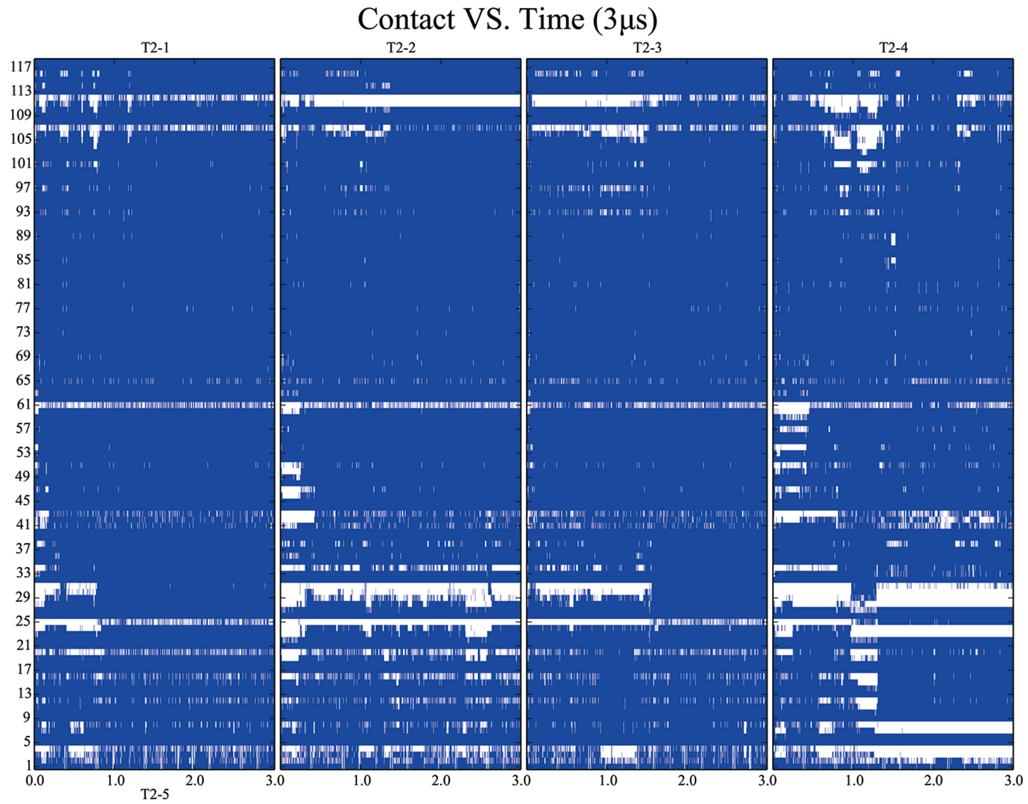

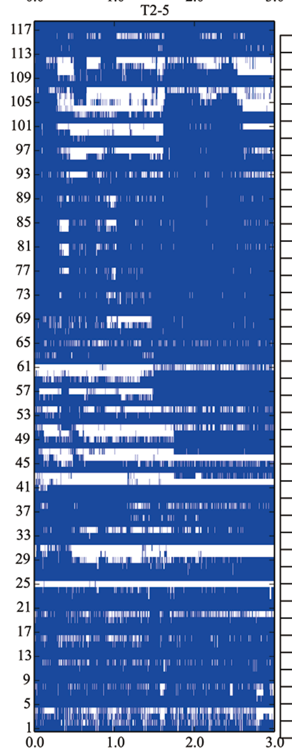

ID	Residue1	Residue2	ID	Residue1	Residue2	ID	Residue1	Residue2
1	1	2	41	12	14	81	23	27
2	1	3	42	12	16	82	24	25
3	1	5	43	12	17	83	24	26
4	1	6	44	13	14	84	24	27
5	2	3	45	13	15	85	24	28
6	2	4	46	13	16	86	25	26
7	2	5	47	13	17	87	25	27
8	2	6	48	14	15	88	25	28
9	3	4	49	14	16	89	25	29
10	3	5	50	14	17	90	26	27
11	3	6	51	14	18	91	26	28
12	3	7	52	15	16	92	26	29
13	4	5	53	15	17	93	26	30
14	4	6	54	15	18	94	27	28
15	4	7	55	16	17	95	27	29
16	4	8	56	16	18	96	27	30
17	5	6	57	16	19	97	27	31
18	5	7	58	17	18	98	28	29
19	5	8	59	17	19	99	28	30
20	5	9	60	17	20	100	28	31
21	6	7	61	17	25	101	28	32
22	6	8	62	18	19	102	29	30
23	6	9	63	18	20	103	29	31
24	6	10	64	19	20	104	29	32
25	6	13	65	19	21	105	29	33
26	7	8	66	20	21	106	29	34
27	7	9	67	20	22	107	29	35
28	7	10	68	20	24	108	30	31
29	7	11	69	20	25	109	30	32
30	7	12	70	21	22	110	30	33
31	7	13	71	21	23	111	30	34
32	8	9	72	21	24	112	30	35
33	8	10	73	21	25	113	31	32
34	8	11	74	22	23	114	31	33
35	9	10	75	22	24	115	32	33
36	9	11	76	22	25	116	32	34
37	10	11	77	22	26	117	33	34
38	10	12	78	23	24	118	34	35
39	11	12	79	23	25			
40	12	13	80	23	26			

Figure S5. The native contact formation vs. simulation time for HP35 with a translation rate of one residue per 2ns. If a native contact is formed it is marked in blue. The native contacts are numbered from N-terminus sequentially. The residue pair related to each native contact is list in the right bottom.
